# Assessment of right ventricular involvement in patients with anti-MDA5 Ab+ DM: a prospectively observational study with cardiac magnetic resonance imaging

**DOI:** 10.3389/fcvm.2025.1558800

**Published:** 2025-05-02

**Authors:** Ling Zhang, Yuli Wang, Jinzhu Dai, Xiaoqi Pu, Anqi Liu, Yifei Ni, Jianping Wang, Jie Du, Yanhong Ren, Xiaoming Shu, Min Liu

**Affiliations:** ^1^ Department of Radiology, China–Japan Friendship Hospital, Beijing, China; ^2^ Department of Radiology, Chinese Academy of Medical Sciences and Peking Union Medical College, Beijing, China; ^3^ Department of Radiology, Capital Medical University, Beijing, China; ^4^ Department of Pulmonary and Critical Care Medicine, China–Japan Friendship Hospital, Beijing, China; ^5^ Department of Rheumatology and Immunology, China–Japan Friendship Hospital, Beijing, China

**Keywords:** MDA5+ DM, right ventricular function, right ventricular long-axis strain (RV-LAS), end-diastolic remodeling index, T1 mapping, T2 mapping

## Abstract

**Background:**

Anti-melanoma differentiation-associated gene 5 dermatomyositis (anti-MDA5 Ab+ DM) is characterized by amyopathic DM with interstitial lung disease. Its impact on the right ventricle remains unclear. We aim to evaluate RV involvement in anti-MDA5 Ab+ DM patients using cardiovascular magnetic resonance (CMR).

**Methods:**

This single-center, prospective cohort study included 43 anti-MDA5 Ab+ DM patients (24 males, mean age = 44.7 ± 11.1 years) and 30 age- and gender-matched healthy controls (18 males, mean age = 44.8 ± 10.4 years). All patients underwent CMR before treatment. RV functional parameters, including ejection fraction (RVEF), end-diastolic/end-systolic remodeling index (RVEDRI/RVESRI), and right ventricular long-axis strain (RV-LAS), and RV and LV T1 and T2 mapping were analyzed. Differences between the two groups were evaluated, and correlations with clinical data were explored.

**Results:**

Anti-MDA5 Ab+ DM patients exhibited a significant decrease in RVEF (45.7 ± 5.9% vs. 52.7 ± 6.6%, *P* < 0.001) and RV-LAS across all techniques. Increased RVESRI (1.38 ± 0.14 vs. 1.29 ± 0.14, *P* = 0.031) indicated RV subclinical dysfunction. The RV and LV blood pool T2 ratio was elevated in patients (0.96 ± 0.02 vs. 0.94 ± 0.03, *P* = 0.007). Patients in the inflammatory marker-positive group exhibited significantly worse RV-LAS compared with the negative group. RV-LAS_Ins/mid_ negatively correlated with hsTnl levels (*r* = −0.34, *P* = 0.026), and ferritin (FER) is moderately positively correlated with RV-LAS_LVapex/peri_ (*r* = 0.487, *P* < 0.001).

**Conclusion:**

RV subclinical dysfunction is common in patients with anti-MDA5 Ab+ DM. RV parameters on CMR such as RV-LAS and RVESRI serve as valuable imaging markers for early detection and risk stratification. These findings underscore the importance of routine cardiac evaluation in anti-MDA5 Ab+ DM.

## 1 Introduction

Idiopathic inflammatory myopathy (IIM) is an autoimmune disease primarily affecting skeletal muscles and various systemic organs, including the skin, lungs, heart, and joints (1). The anti-melanoma differentiation-associated gene 5 (anti-MDA5) antibody was first reported in 2005 (2) as the anti-clinically amyopathic dermatomyositis-140 (anti-CADM-140) antibody. It has been strongly associated with rapidly progressive interstitial lung disease (RP-ILD). Previous studies (3, 4) have shown that 6%–75% of patients with dermatomyositis (DM) experience concomitant cardiac disorders, including heart failure, coronary artery disease, and conduction abnormalities. A previous study found that left ventricular (LV) involvement is common in anti-MDA5 Ab+ DM patients (5).

Right ventricular (RV) dysfunction represents an important independent predictor of adverse outcomes in many cardiovascular diseases (6, 7). RV function is a key prognostic factor in patients with systemic lupus erythematosus (SLE), with mortality closely linked to RV function. Early detection of subclinical RV dysfunction is crucial for developing treatment strategies and improving prognosis in SLE patients (8, 9). However, compared with the left ventricle, the right ventricle has a more irregular shape and movement, making it challenging to accurately assess RV function, especially subclinical dysfunction. In patients with anti-MDA5 Ab+ DM, whether the right ventricle is involved remains unknown.

Cardiovascular magnetic resonance (CMR) is the gold standard for assessing LV and RV function and tissue characteristics (10). In studies by Arenja et al. (11) and Shang et al. (12), long-axis strain (LAS) has been proposed as an accurate and simple measure of RV function in non-ischemic dilated cardiomyopathy and type 2 diabetes mellitus. Zhang et al. (13) reported that the CMR-derived right ventricular end-systolic modeling index (RVESRI) is a simple and reproducible metric in assessing right ventricular dysfunction and hemodynamics in patients with chronic thromboembolic pulmonary hypertension. Moreover, Saini et al. (14) showed that blood T2 is sensitive to the level of blood oxygenation, and quantitative T2 mapping is a novel, non-invasive method to estimate blood O_2_ saturation. Emrich et al. (15) reported that RV and LV blood pool T2 ratio can qualify left-to-right shunts in patients with known L–R shunt diseases. Deng et al. (16) introduced RV and LV blood pool T2 ratio on T2 mapping could be an additional CMR imaging marker that may assist in evaluating the severity of right ventricular dysfunction and hemodynamics in patients with pulmonary hypertension.

It is unclear whether RV functional metrics and RV and LV blood pool T2 ratio on CMR are affected in anti-MDA5 Ab+ DM patients. Therefore, this study aimed to evaluate RV involvement in anti-MDA5 Ab+ DM patients using CMR.

## 2 Methods

### 2.1 Study design and participants

**Figure 1:**
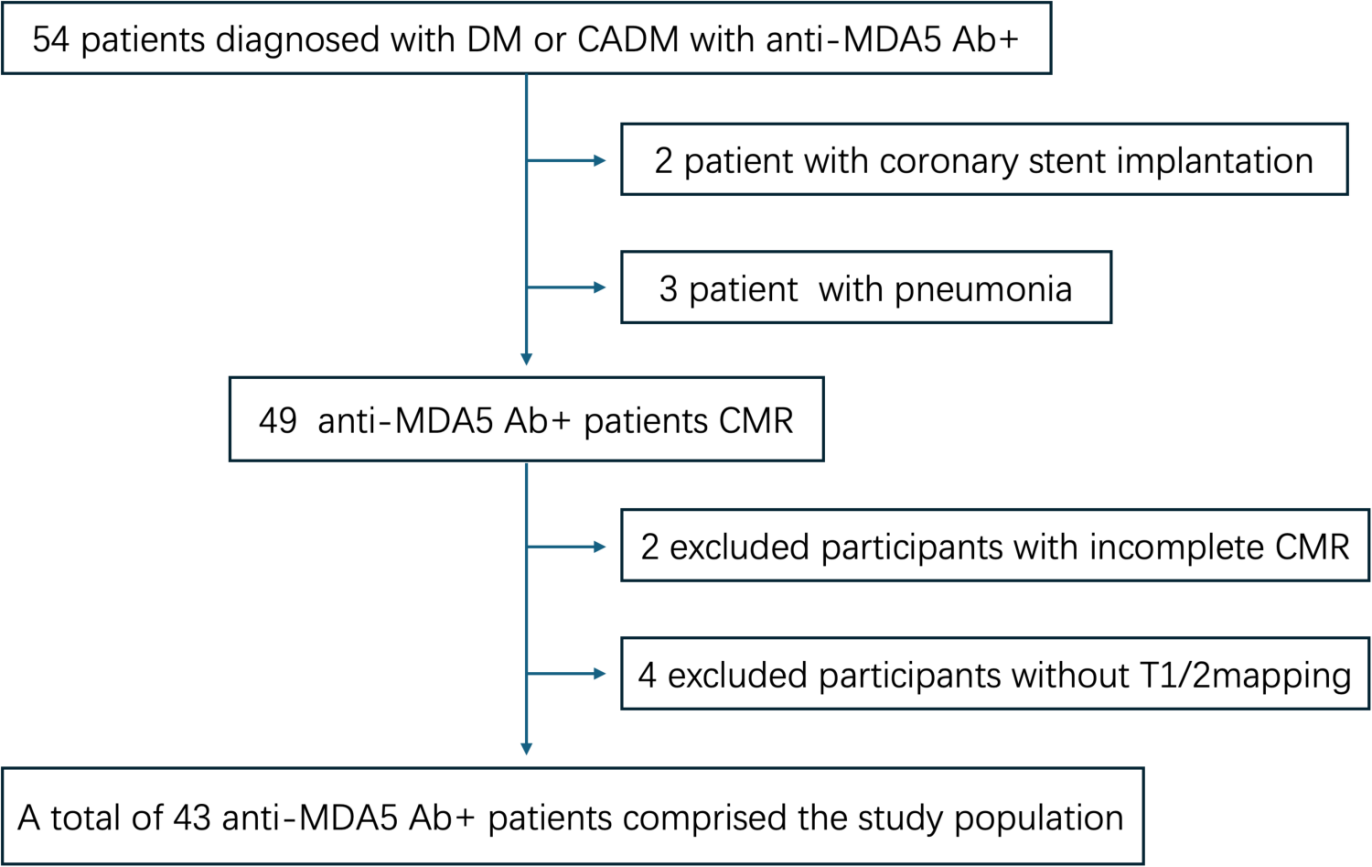
The flowchart of the study.

This single-center, prospective cohort study was performed according to the Declaration of Helsinki and was approved by the Ethics Committee of our hospital (approval number: 2021-KY-060). All participants signed informed consent before the CMR scan. Patients who were diagnosed with anti-MDA5 Ab+ DM at our hospital between May 2021 and May 2024 were included. DM was diagnosed based on the 239th European Neuromuscular Centre International Workshop (17). Meanwhile, age- and gender-matched volunteers with normal electrocardiogram and echocardiography findings were grouped into the healthy controls (HC). All patients underwent non-contrast and contrast-enhanced CMR, and all HC only underwent non-contrast CMR. Exclusion criteria were as follows: (1) CMR in poor image quality due to breath artifacts or severe arrhythmia; (2) patients with coronary stent implantation or coronary artery bypass grafting; (3) patients with proven or suspected ongoing infection; (4) patients with the history of congenital heart disease, coronary heart disease, or cardiomyopathy or malignant tumor; (5) patients with other types of connective tissue disease or myositis-specific autoantibodies; and (6) patients with hypothyroidism or renal or hepatic dysfunction. The flowchart of this study is shown in Figure 1.

### 2.2 Cardiac MR protocol

All patients underwent CMR on a 1.5 Tesla clinical MR scanner (MAGNETOM Aera, Siemens Healthcare, Erlangen, Germany) with an 18-channel phased-array surface coil. All images were acquired with retrospective electrocardiographic gating in the end-expiratory breath hold. The CMRI scanning protocol included: (1) conventional sequences, i.e., short-axis and long-axis cine and T2-weighted imaging (T2WI); (2) tissue mapping sequences, i.e., native T1 mapping and T2 mapping; and (3) late gadolinium enhancement. The stack of short-axis slices covered the left ventricle (LV) from the apex to the mitral annulus.

Standard contiguous short-axis slices covering both ventricles from base to apex and long-axis four-chamber cine images were acquired with the balanced steady-state free precession sequence. The typical acquisition parameters included repetition time (TR) = 34.5 ms, echo time (TE) = 1.1 ms, FOV = 360 mm × 256 mm, flip angle (FA) = 50–60°, slice thickness = 6 mm, in-plane spatial resolution 1.8 × 1.8 mm^2^, temporal resolution 40 ms, and 25 reconstructed cardiac phases. Native T1 mapping was performed using electrocardiograph-gated, diastole-triggered, single-shot modified Look–Locker IR sequence with protocol 3 (3 s) 3 (3 s) 5, acquiring seven images in 17 heartbeats, with TE = 1.2 ms, TR = 2.8 ms, FOV = 360 mm  × 360 mm, matrix = 128 × 128, FA = 35°, bandwidth = 100 kHz, slice thickness = 8 mm, and slice gap = 0 mm. T2 mapping was generated using a double IR fast spin echo sequence with four different TE (11, 33, 55.1, and 77.1 ms) for a total echo train length = 16, TR = 629 ms, FA = 90°, matrix = 160 × 160, bandwidth = 83.33 kH, slice thickness = 8 mm, and slice gap = 3 mm.

### 2.3 Right ventricular function analysis

CMR images in Digital Imaging and Communications in Medicine (DICOM) format were transferred to the syngo.via workstation (Siemens Healthcare Sector, Forchheim, Germany). The endocardial and epicardial contours of all short-axis slices encompassing the right ventricle (RV) during the end-diastolic (ED) and end-systolic (ES) phases were manually delineated on CardiacFunction by a cardiovascular radiologist with 10-years of experience. This process allowed for the calculation of the RV end-diastolic volume index (EDVI), end-systolic volume index (ESVI), stroke volume index (SVI), ejection fraction (EF), and RV cardiac index (RVCI).

### 2.4 RV-LAS measurement

**Figure 2:**
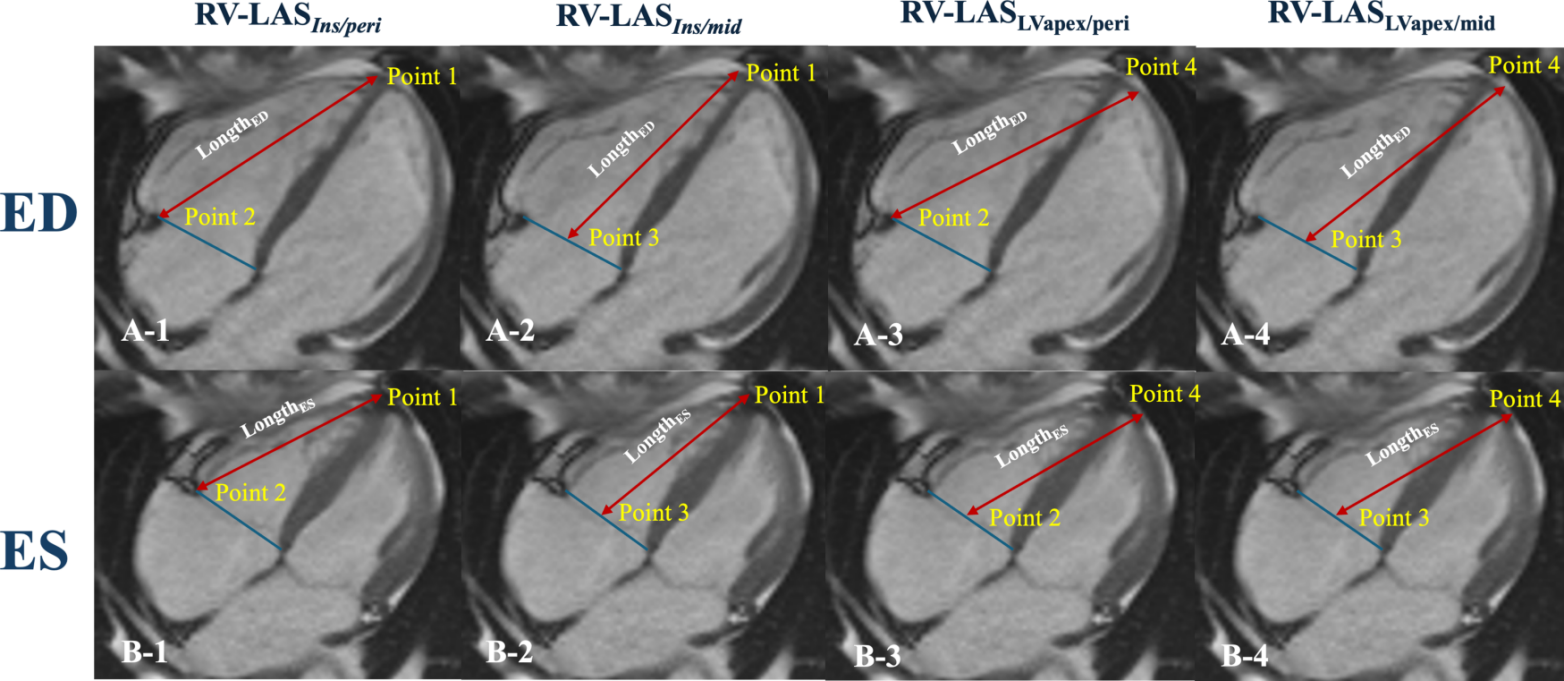
Schematic diagram for right ventricular longitudinal function measurements. Illustration of the four different LAS measurements **(A,B)**. RV-LA_Sins/peri_: Point 1 is the insertion point between the RV and LV, and Point 2 is the lateral insertion of the tricuspid valve; Length_ED_, Point 1 to Point 2 **(A-1)**; Length_ES_, Point 1 to Point 2′ **(B-1)**. RV-LA_Sins/mid_: Point 3 is the middle of the tricuspid valve ring; Length_ED_, Point 1 to Point 3 **(A-2)**; Length_ES_, Point 1 to Point 3 **(B-2)**. RV-LAS_LVapex/peri_: Point 4 is the epicardial border of the LVapex; Length_ED_, Point 4 to Point 2 **(A-3)**; Length_ES_, Point 4 to Point 2 **(B-3)**. RV-LAS_LVapex/mid_: Length_ED_, Point 4 to Point 3 **(A-4)**; Length_ES_, Point 4 to Point 3 **(B-4)**.

Based on Arenja et al. (11), RV-LAS was measured on four-chamber images in the assessment of the displacement of the tricuspid annulus (Figure 2) by a cardiovascular radiologist with 10 years of experience:

I. RV-LAS_Ins/peri_: The length between the epicardial border at the insertion point between the RV and LV and the lateral insertion of the tricuspid valve was measured at both end-systole and end-diastole.
II. RV-LAS_Ins/mid_: The length between the epicardial border at the insertion point between the RV and LV and the midpoint of a line connecting the origins of the tricuspid valve leaflets was measured at both end-systole and end-diastole.
III. RV-LAS_LVapex/peri_: The length between the epicardial border of the LV apex and the lateral insertion of the tricuspid valve was measured at both end-systole and end-diastole.
IV. RV-LAS_LVapex/mid_: The length between the epicardial border of the LV apex and the midpoint of a line connecting the origins of the tricuspid valve leaflets was measured at both end-systole and end-diastole.

The value for RV-LAS was calculated as follows:

$$\mathrm{RV}-\mathrm{LAS}=\frac{\mathrm{lengt}h_{\mathrm{end}\text{-}\mathrm{systole}}-\mathrm{lengt}h_{\mathrm{end}\text{-}\mathrm{diastole}}}{\mathrm{lengt}h_{\mathrm{end}\text{-}\mathrm{diastole}}}\times 100\text{\%}$$

### 2.5 Right ventricular remodeling index measurement

**Figure 3:**
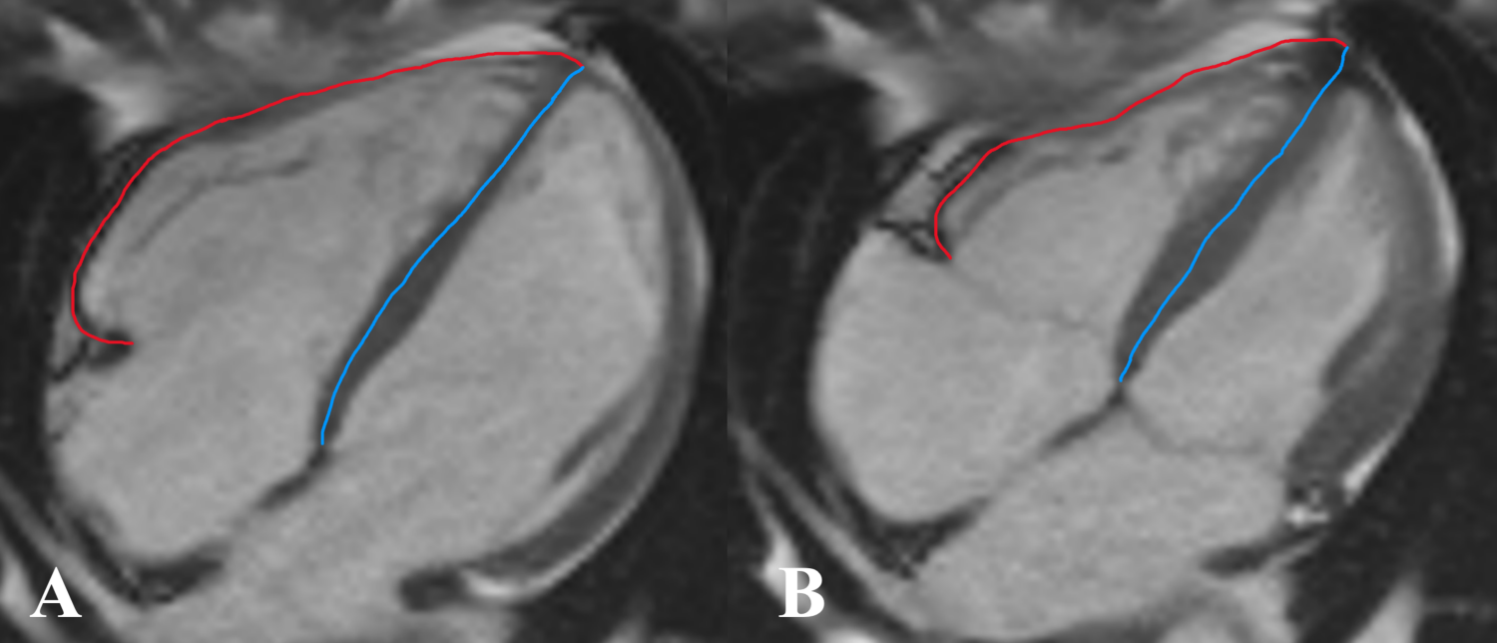
Measurement of right ventricular end-diastolic remodeling index (RVEDRI) **(A)** and right ventricular end-systolic remodeling index (RVESRI) **(B)** on the four-chamber cine image of cardiac magnetic resonance imaging (CMR). RVEDRI and RVESRI were the ratios of lateral free wall length (red line) to interventricular septal height (blue line).

According to Zhang et al. (11), the right ventricular end-diastolic remodeling index (RVEDRI) and RVESRI are the ratio of the lateral free wall length to septal wall height in the end-systolic phase and end-diastolic phase which was measured on the long-axis four-chamber cine image of CMR (Figure 3) by a cardiovascular radiologist.

### 2.6 Ventricular blood pool T1 and T2

**Figure 4:**
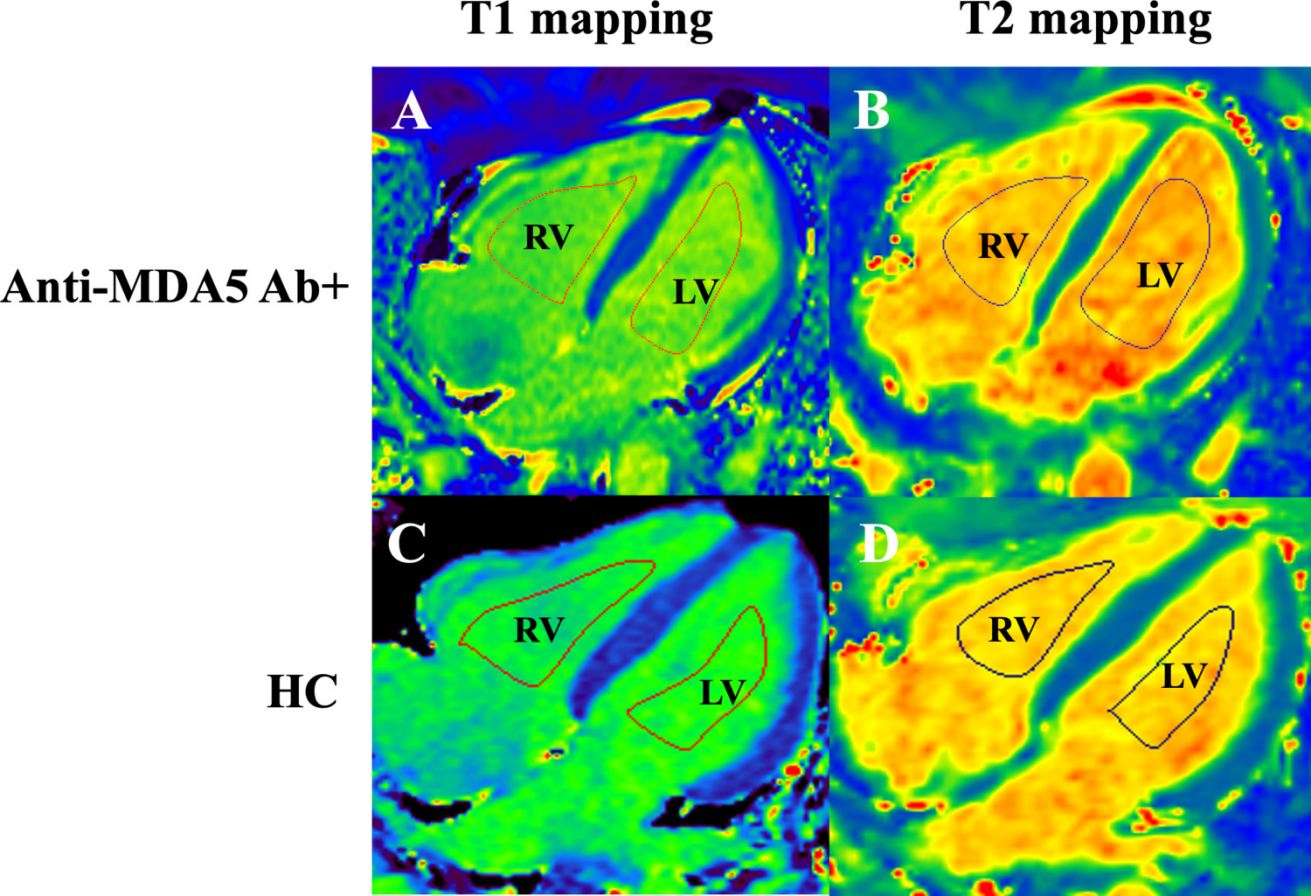
Measurement of T1 and T2 relaxation times in the right (RV) and left (LV) ventricular blood pools in the four-chamber cardiac view in anti-MDA5 Ab+ DM patients **(A,B)** and HC (healthy controls) **(C,D)**.

Based on Deng et al. (14), T1 and T2 values of the RV and LV blood pools were respectively measured on the four-chamber slices of the T1 map and T2 map with syngo.via workstation (Siemens Healthcare Sector, Forchheim, Germany) by two cardiovascular radiologists. Regions of interest (ROIs) for the RV and LV blood pools were delineated along the endomyocardium on both the T1 map and T2 map (Figure 4). To minimize bias, the T1 and T2 values were independently measured by two cardiovascular radiologists who were blinded to clinical information. Additionally, one observer repeated the analysis of the RV and LV blood pool T1 and T2 values after a 4-week interval. The ratios of RV to LV blood pool T1 (RVT1/LVT1 ratio) and T2 (RVT2/LVT2 ratio) were calculated as the mean of three measurements (Figure 4).

### 2.7 Statistical analysis

SPSS (SPSS 29.0, SPSS Inc., Chicago, IL, USA), MedCalc (version 15.0, Mariakerke, Belgium), and GraphPad Prism (version 10.4.1; GraphPad Software) were used for statistical analysis and plotting. Data distribution was assessed for normality using the normality test and distribution curve before further statistical analysis. Data are presented as the mean ± SD. To compare anti-MDA5 Ab+ DM patients with normal controls, independent *t*-tests were used for normally distributed data, Mann–Whitney *U* tests for non-normally distributed data, and chi-square tests for categorical variables. Correlation analysis was performed to assess the relationships between variables. Pearson's correlation coefficient was used for normally distributed data, while Spearman's rank correlation was used for non-normally distributed data. *P* < 0.05 was considered statistically significant.

## 3 Results

### 3.1 Patients characteristics

A total of 73 participants (42 males, mean age = 44.7 ± 10.7 years old), including 43 anti-MDA5 Ab+ patients (24 males, mean age = 44.7 ± 11.1 years old) and 30 healthy control subjects (18 males, mean age = 44.8 ± 10.4 years old) were enrolled in this study. The demographic and clinical data between the anti-MDA5 Ab+ and HC groups are summarized in Table 1.

**Table 1:** Demographic and clinical information of all participants.

Characteristic	Anti-MDA5 Ab+ (*N* = 43)	Healthy controls (*N* = 30)	*χ*^2^/*t* (*P*)
Male, *N* (%)	24 (55.8%)	18 (60%)	*χ*^2^ = 0.069 (0.764)
Age (years)	44.7 ± 11.1	44.8 ± 10.4	−0.045 (0.964)
BMI (kg/m^2^)	21.4 ± 2.9	22.3 ± 3.4	−0.051 (0.269)
SBP (mmHg)	122.2 ± 14.4	124.1 ± 10.6	−0.925 (0.139)
DBP (mmHg)	79.3 ± 10.6	81.4 ± 10.3	−1.050 (0.677)
D-dimer (mg/L)	0.70 ± 0.14	0.24 ± 0.27	5.222 (<0.001)**
hsTnl (ng/ml)	0.0105 ± 0.0306	0.0069 ± 0.0025	0.785 (0.437)
Cytokines (+), *N* (%)	22 (51%)	–	
ESR (mm/h)	71 ± 16.6	–	
FER (ng/ml)	619.0 ± 686.3	–	
LDH (U/L)	295.7 ± 144	–	
NT-pro-BN (pg/ml)	101.7 ± 80.8	–	
ILD, *N* (%)	43 (100%)	–	

SBP, systolic blood pressure; DBP, diastolic blood pressure; ESR, erythrocyte sedimentation rate FER, ferritin; LDH, lactate dehydrogenase; ILD, interstitial lung disease.

Data are reported as counts and percentages for categorical data and mean ± standard deviation (M ± SD) (for normal distribution) for continuous data.

* *P* < 0.05. **P < 0.001.

For MDA5+ DM patients, laboratory tests also included cytokines (IL-6, IL-8, IL-10, IL-1β, TNF-α, IFN-γ, etc.), inflammatory markers [erythrocyte sedimentation rate (ESR), ferritin (FER), and lactate dehydrogenase (LDH)], and NT-proBNP. All MDA5+ patients had chest CT scans that indicated the presence of ILD, as shown in Table 1. As shown in Table 2, the main pulmonary artery (MPA) diameter and ascending aortic diameter ratio (MPA/Aao ratio) are comparable between the anti-MDA5 Ab+ and HC groups, and there were no significant statistical differences between the two groups.

**Table 2:** RV-LAS, ventricular blood pool T1 and T2, and RV function for all participants.

Characteristic	Anti-MDA5 Ab+ (*N* = 43)	Healthy controls (*N* = 30)	*χ*^2^/*t* (*P*)
RV function			
EF (%)	45.7 ± 5.9	52.7 ± 6.6	−4.767 (<0.001)**
EDV (ml)	119.4 ± 49.2	120.6 ± 26.5	−0.135 (0.893)
ESV (ml)	65.1 ± 28.6	57.1 ± 15.4	1.529 (0.131)
SV (ml)	54.8 ± 22.9	63.6 ± 15.5	1.969 (0.53)
CO (L/min)	4.3 ± 1.8	4.7 ± 1.4	−0.973 (0.334)
EDVI (ml/m^2^)	68.1 ± 25.9	61.6 ± 10.8	1.467 (0.148)
ESVI (ml/m^2^)	37.7 ± 15.6	29.1 ± 6.7	3.217 (0.002)*
SVI (ml/m^2^)	30.9 ± 11.6	32.5 ± 6.7	−0.694 (0.49)
CI (l/min/m^2^)	2.5 ± 0.9	2.4 ± 0.6	0.459 (0.648)
RV remodeling index			
RVEDRI	1.59 ± 0.13	1.53 ± 0.14	1.641 (0.052)
RVESRI	1.38 ± 0.14	1.29 ± 0.14	2.899 (0.031)*
RV-LAS			
RV-LAS_Ins/peri_ (%)	−24.8 ± 7.8	−26.8 ± 5.6	1.251 (0.026)*
RV-LAS_Ins/mid_ (%)	−24.2 ± 7.8	−27.1 ± 5.5	1.892 (0.019)*
RV-LAS_LVapex/peri_ (%)	−17.1 ± 4.9	−20.4 ± 5.6	2.489 (0.016)*
RV-LAS_LVapex/mid_ (%)	−16.9 ± 4.3	−19.3 ± 5.1	2.839 (0.006)*
Ventricular blood pool T1/T2 mapping			
RVT1 (ms)	1,536.3 ± 83.3	1,465.1 ± 99.3	3.220 (0.002)*
LVT1 (ms)	1,594.6 ± 77.4	1,553.2 ± 77.3	2.251 (0.028)*
RVT1/LVT1 ratio	0.96 ± 0.02	0.94 ± 0.03	2.819 (0.007)*
RVT2 (ms)	137.6 ± 16.2	131.1 ± 12.7	1.944 (0.056)
LVT2 (ms)	158.8 ± 23.1	154.3 ± 15.3	0.995 (0.323)
RVT2/LVT2 ratio	0.88 ± 0.12	0.85 ± 0.07	1.133 (0.261)
PH Markers			
MPA (mm)	26.5 ± 4.2	24.4 ± 2.6	1.395 (0.167)
MPA/Aao ratio	0.912 ± 0.113	0.800 ± 0.106	1.618 (0.055)

RV, right ventricle; EF, ejection fraction; EDV, end-diastolic volume; ESV, end-systolic volume; EDVI, end-diastolic volume index; ESVI, end-systolic volume index; SV, stroke volume; CO, cardiac output; SVI, stroke volume index; CI, cardiac index; MPA, main pulmonary artery diameter; MPA/Aao ratio, main pulmonary artery diameter and ascending aortic diameter ratio.

Data are reported as counts and percentages for categorical data and mean ± standard deviation (M ± SD) (for normal distribution) for continuous data.

* *P* < 0.05. **P < 0.001.

### 3.2 RV function and RV remodeling index on CINE

As shown in Table 2, RVEF in the anti-MDA5 Ab+ DM group significantly reduced (45.7 ± 5.9% vs. 52.7 ± 6.6%, *P* < 0.001) in comparison with the HC group. In contrast, RVESVI significantly increased in the anti-MDA5 Ab+ DM group (37.7 ± 15.6 ml/m^2^ vs. 29.1 ± 6.7 ml/m^2^, *P* = 0.002). There was a significant difference in RVEDRI between the anti-MDA5 Ab+ DM and HC groups (1.38 ± 0.14 vs. 1.39 ± 0.14, *P* = 0.031). However, there were no statistically significant differences in the remaining RV function and RVESRI between the two groups.

### 3.3 Analysis of RV-LAS between the two group

**Figure 5:**
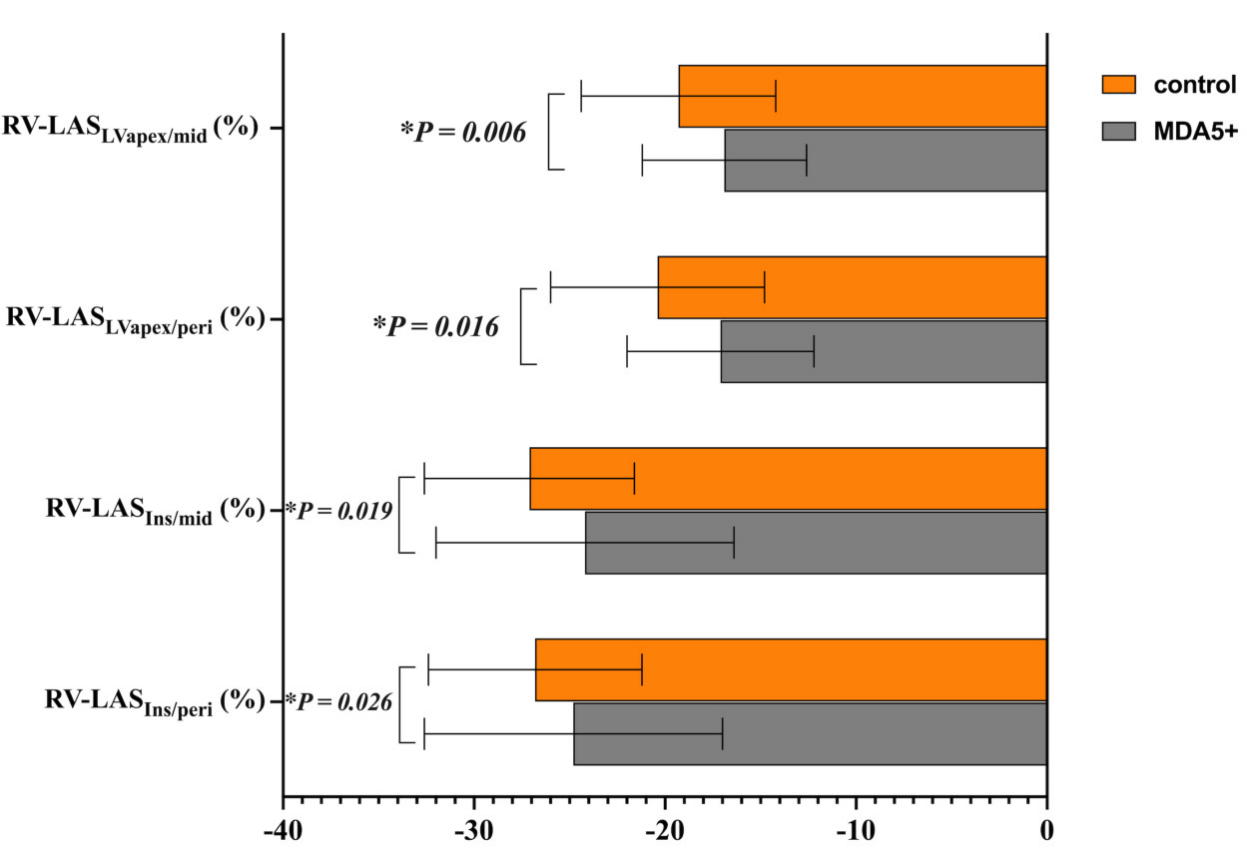
RV long-axis strain (RV-LAS) in anti-MDA5 Ab+ DM patients and healthy controls. *P < 0.05 in the comparison between patients and controls.

Regarding all four techniques of RV-LAS (Figure 5), anti-MDA5 Ab+ DM patients had higher RV-LAS compared with HC (RV-LAS_Ins/peri_, −24.8 ± 7.8% vs. −26.8 ± 5.6%, *P* = 0.026; RV-LAS_Ins/mid_, −24.2 ± 7.8% vs. −27.1 ± 5.5%, *P* = 0.019; RV-LAS_LVapex/peri_, −17.1 ± 4.9% vs. −20.4 ± 5.6%, *P* = 0.016; RV-LAS_LVapex/mid_, −16.9 ± 4.3% vs. −19.3 ± 5.1%, *P* = 0.006).

### 3.4 Comparison of ventricular blood pool T1 and T2 values

**Figure 6:**
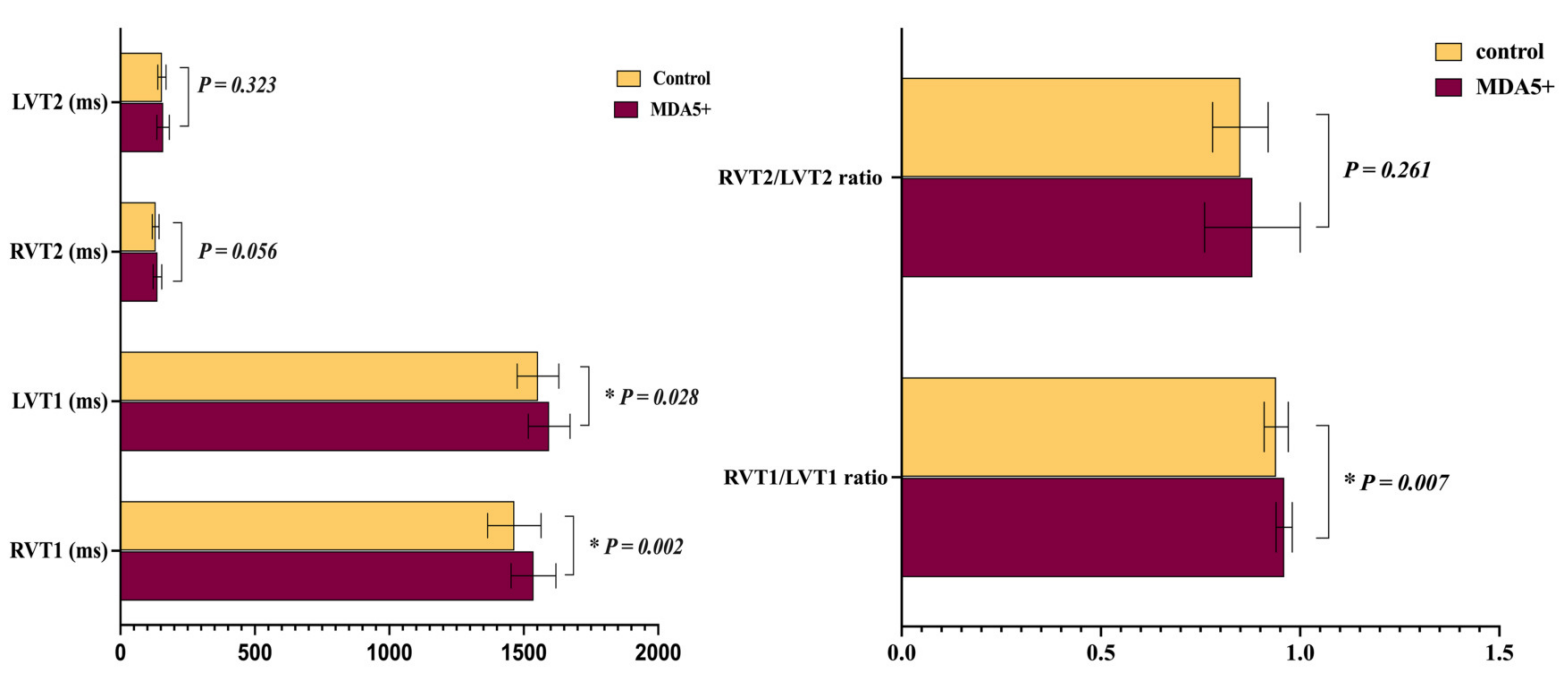
Ventricular blood pool T1 and T2 values in anti-MDA5 Ab+ DM patients and healthy controls. *P < 0.05 in the comparison between patients and controls.

As shown in Figure 6, RVT1, LVT1, and RVT1/LVT1 ratio in anti-MDA5 Ab+ DM patients were higher than in the control group (RVT1, 1,536.3 ± 83.3 ms vs. 1,465.1 ± 99.3 ms, *P* = 0.002; LVT1, 1,594.6 ± 77.4 ms vs. 1,553.2 ± 77.3 ms, *P* = 0.028; RVT1/LVT1 ratio, 0.96 ± 0.02 vs. 0.94 ± 0.03, *P* = 0.007), but there were no significant differences in RVT2, LVT2, and RVT2/LVT2 ratio between the anti-MDA5 Ab+ DM and HC groups.

### 3.5 Correlation of RV parameters and clinical markers

**Figure 7:**
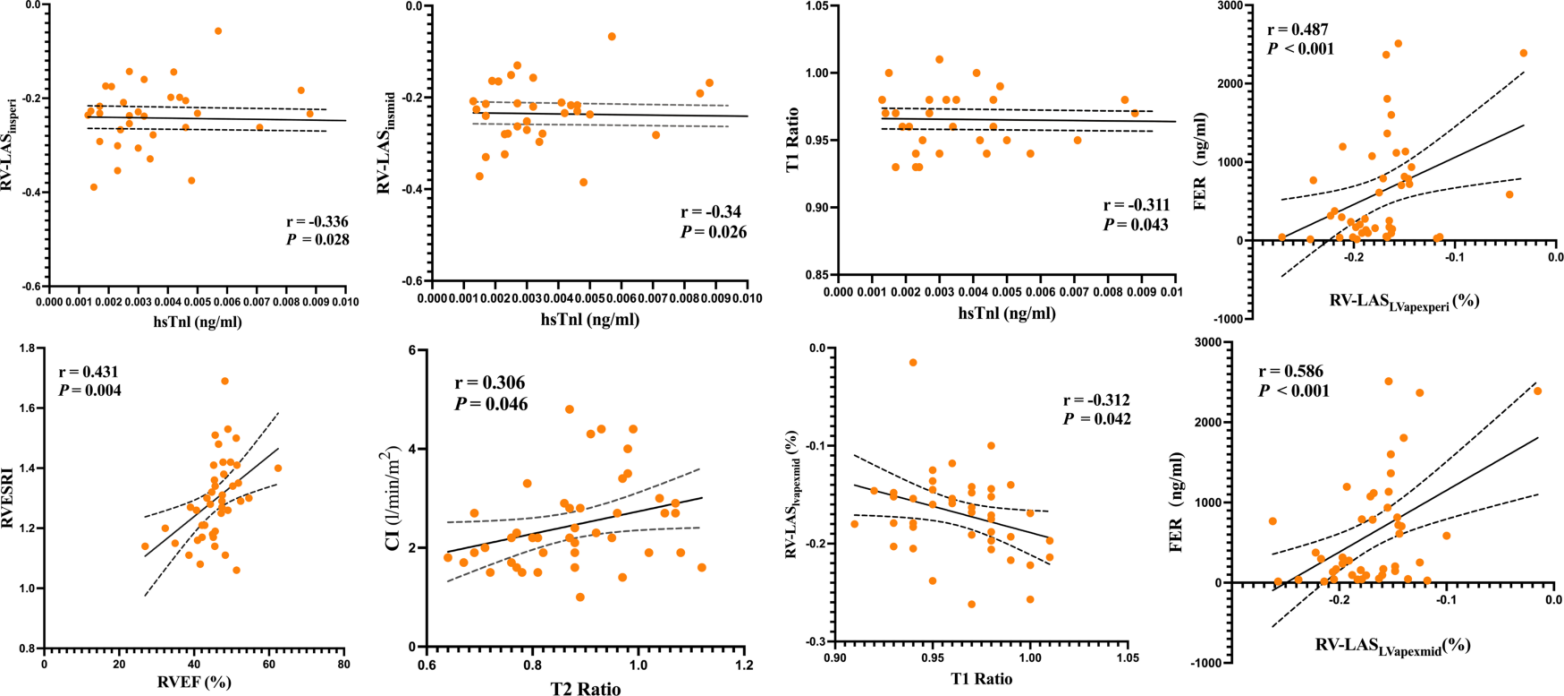
Linear correlation analysis among RVESRI, RV-LAS, ventricular blood pool T1 and T2 values, RV function, and biochemical examinations.

In the anti-MDA5 Ab+ patients, RV-LAS_Ins/mid_ (*r* = −0.34, *P* = 0.026), RV-LAS_Ins/peri_ (*r* = −0.336, *P* = 0.028), and T1 ratio (*r* = −0.311, *P* = 0.043) negatively correlated with hsTnl levels. RV-LAS_LVapex/mid_ demonstrated a moderate negative correlation with T1 ratio (*r* = −0.312, *P* = 0.042), RV/LV T2 ratio was moderately positive correlated with CI (*r* = 0.306, *P* = 0.046) (Figure 7), and RVESRI was moderately positive correlated with RVEF (*r* = 0.431, *P* = 0.004). Both RV-LAS_LVapex/peri_ (*r* = 0.487, *P* < 0.001) and RV-LAS_LVapex/mid_ (*r* = 0.586, *P* < 0.001) positively correlated with FER. There were no significant correlations between other right ventricular function parameters and clinical data.

**Figure 8:**
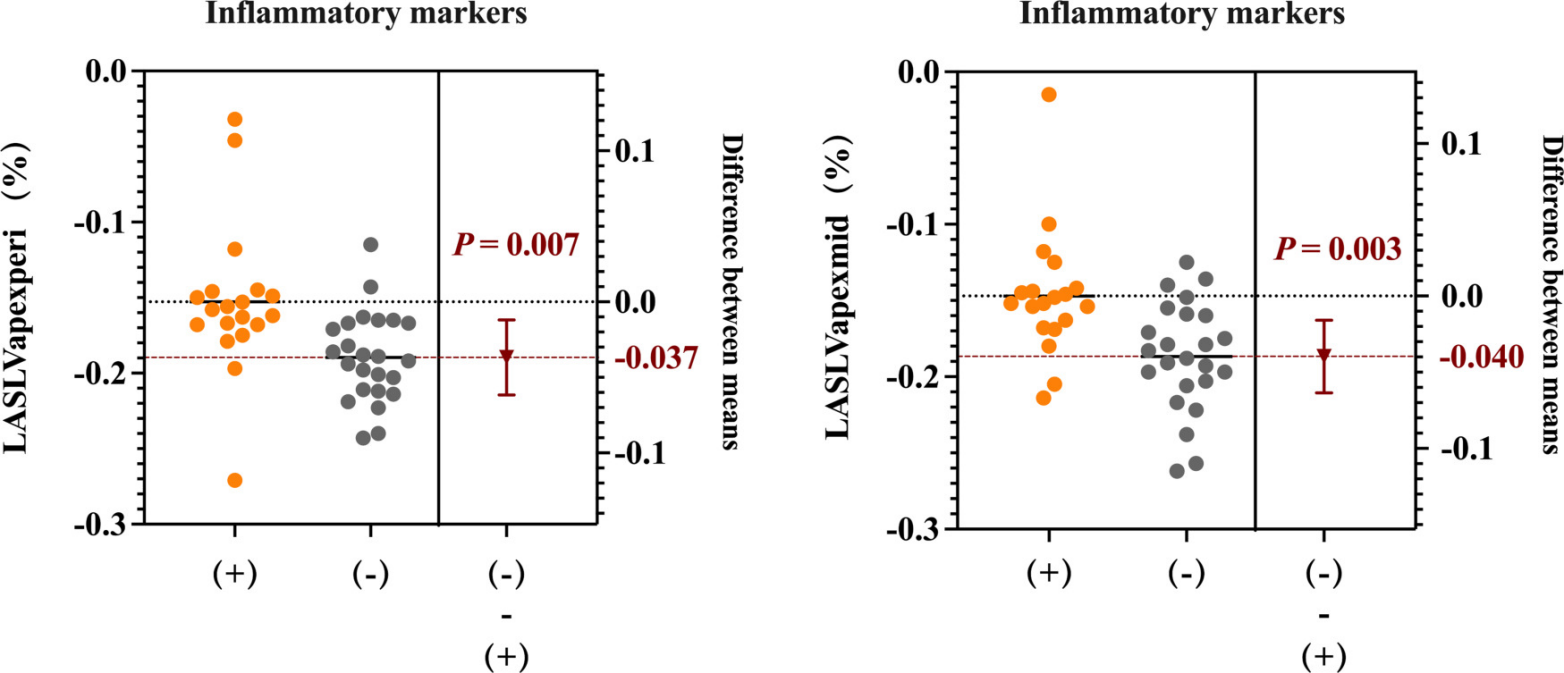
RV long-axis strain (RV-LAS) (RV-LAS_LVapex/peri_ and RV-LAS_LVapex/mid_) in patients between the inflammatory marker-positive group and the negative group.

The 43 patients with MDA5+ DM were stratified into subgroups based on predefined biomarker abnormality thresholds: (1) cytokine-abnormal group (23.3%, 10/43) vs. cytokine-normal group (76.7%, 33/43); (2) inflammatory marker-positive group (48.8%, 21/43) vs. inflammatory marker-negative group (51.2%, 22/43); and (3) NT-proBNP-normal group (20.9%, 9/43) vs. NT-proBNP-abnormal group (79.1%, 34/43). Then, we compared the right ventricular parameters within each group. The inflammatory marker-positive group exhibited significantly worse RV-LAS compared with the negative group (RV-LAS_LVapex/peri_, −15.4 ± 4.9% vs. −19.0 ± 3.0%, *P* = 0.007; RV-LAS_LVapex/mid_, −14.9 ± 4.1% vs. −18.7 ± 3.7%, *P* = 0.003) (Figure 8). However, no significant statistical differences in right ventricular function parameters were observed between subgroups stratified by cytokine status or NT-proBNP levels.

### 3.6 The prognostic evaluation value of RV-LAS and ventricular blood pool T1 and T2 values

**Figure 9:**
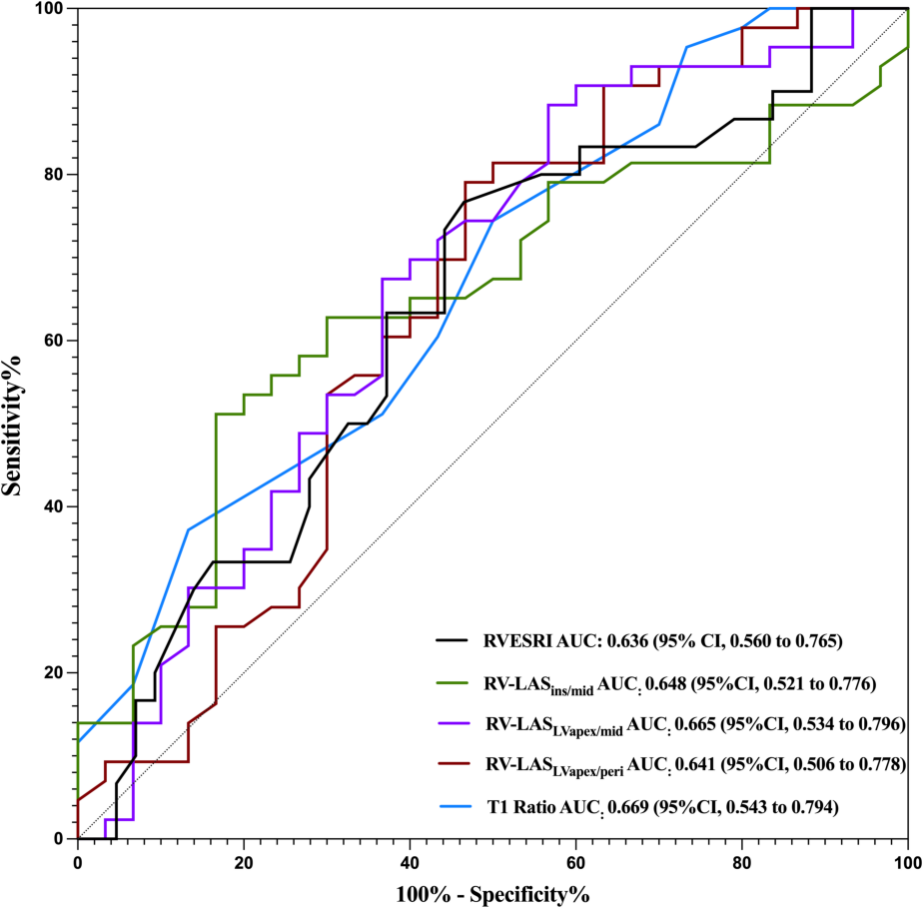
ROC curves for different parameters of RV-LAS and ventricular blood pool T1 and T2 values. The AUCs for distinguishing the HCs from the MDA5+ group are 0.636 for RVESRI, 0.648 for RV-LAS_Ins/mid_, 0.665 for RV-LAS_LVapex/peri_, and 0.641 for RV-LAS_LVapex/mid_ and 0.669 for T1 ratio.

Figure 9 shows the diagnostic performance of T1 ratio, RV-LAS_Ins/mid_, RV-LAS_LVapex/peri_, and RV-LAS_LVapex/mid_ to discriminate patients from controls. The optimal cutoff values for distinguishing between the healthy control group and anti-MDA5 Ab+ DM patients were determined using ROC curve analysis. For the T1 ratio with a cutoff value identified as 0.95, corresponding to a sensitivity of 74.4% and a specificity of 50%, the AUC was 0.669. For RV-LAS_Ins/mid_ with a cutoff value of −23.1%, the AUC, sensitivity, and specificity were 0.648, 51.2%, and 83.3%, respectively. For RV-LAS_LVapex/mid_ with a cutoff value of −20.2%, the AUC, sensitivity, and specificity were 0.665, 79.1%, and 53.3%, respectively. For RV-LASLV_apex/peri_ with a cutoff value of −21.5%, the AUC, sensitivity, and specificity were 0.641, 88.4%, and 43.3%, respectively. For RVESRI with a cutoff value of 1.34, the AUC, sensitivity, and specificity were 0.636, 76.7%, and 53.5%, respectively.

## 4 Discussion

In this prospective study, we provide the first evidence of prevalent RV subclinical dysfunction in anti-MDA5 Ab+ DM patients on CMR with RV-LAS and RVESRI emerging as sensitive markers for early detection. The derived thresholds—T1 ratio > 0.95, RV-LAS_Ins/mid_ < −23.1%, RV-LAS_LVapex/peri_ < −20.2%, RV-LAS_LVapex/mid_ < −21.5%, and RVESRI >1.34—may serve as robust discriminators of RV impairment for anti-MDA5 Ab+ DM patients in clinical practice. These findings extend prior observations of RV dysfunction in systemic sclerosis (13, 18) and establish anti-MDA5+ DM as a distinct entity warranting dedicated cardiac surveillance.

Cardiac involvement in DM arises from chronic inflammatory exposure, driving microvascular injury, fibrosis, and chamber remodeling. The affected cardiac components include the valves, conduction system, myocardium, endocardium, pericardium, and pulmonary and coronary arteries (19). RV dysfunction is an underrecognized but significant contributor to poor outcomes in systemic autoimmune diseases, including anti-MDA5 Ab+ DM. The current study indicated that, compared with HC, anti-MDA5 Ab+ DM patients exhibited reduced RVEF and increased end-systolic volume index RVESVI, indicating impaired systolic function.

According to our study, anti-MDA5 Ab+ DM patients showed a significant increase in RVESVI values. Compared to other remodeling indices such as RV sphericity, RVESRI has been shown to better predict outcomes in adult PAH, offering advantages in simplicity and reproducibility (20). Notably, RVESRI reflects not only RV dimensions but also longitudinal systolic RV function. RV end-systolic dimensions provide information about both RV size and function (21). In the ROC analysis for detecting anti-MDA5 Ab+ DM patients, RVESRI demonstrated strong performance and was found to correlate with systolic RV function (RVEF).

RV-LAS, a validated marker of RV contractility in conditions ranging from type 2 diabetes to non-ischemic cardiomyopathy (11, 16), was consistently reduced in anti-MDA5+ DM patients, confirming subclinical RV dysfunction. The inverse correlation between RV-LAS and hsTnI suggests shared pathways linking subtle myocardial injury to impaired RV mechanics. Notably, patients with elevated inflammatory markers displayed significantly worse RV-LAS than those without, with FER showing a moderate correlation to strain deterioration. This aligns with established mechanisms where inflammation exacerbates myocardial damage—evidenced by post-interventional biomarker rises, mechanistically, inflammatory cytokines exacerbate ischemia–reperfusion injury by amplifying oxidative stress and promoting fibrotic remodeling (22). In DM, hyperferritinemia (>1,500 ng/ml) independently predicts mortality (23), likely through iron-mediated Fenton reaction-driven fibrosis (24). The synergy between cytokine-induced oxidative stress and iron toxicity may accelerate myocardial stiffening, underscoring the need for early detection via sensitive tools like RV-LAS.

Systemic microvascular dysfunction is a recognized feature in various rheumatic and autoimmune diseases (25). In anti-MDA5 Ab+ DM patients, systemic microvascular dysfunction is particularly significant (26). This condition is associated with RP-ILD which can lead to respiratory failure. RP-ILD is a hallmark of anti-MDA5 Ab+ DM and imposes significant hemodynamic stress on the pulmonary circulation. The elevation in pulmonary vascular resistance leads to increased RV afterload, initiating compensatory remodeling and hypertrophy (27). The correlations observed between RV-LAS and hsTnl and FER in this study suggest a link between subclinical myocardial injury and impaired RV contractility.

Moreover, in patients with anti-MDA5+ DM, ILD-associated pulmonary hypertension (PH) may drive right ventricular pressure overload, while concurrent direct myocardial injury mediated by autoimmune mechanisms could coexist as parallel pathological contributors. In our study, MPA and MPA/Aao ratio were similar between the anti-MDA5 Ab+ group and HC group, indicating subclinical RV dysfunction may be mainly attributed to direct myocardial injury mediated by autoimmune mechanisms. The significant reductions in RV function underscores the potential prognostic value of these markers in predicting adverse outcomes. Future studies should explore whether incorporating these ventricular function parameters and T1/T2 imaging into routine clinical practice can improve risk stratification and prognosis in this population.

### 4.1 Limitations

Several limitations should be acknowledged. First, only mild patients without respiratory failure in the relatively small sample size limits the generalizability of the findings. Larger, multicenter studies are needed to validate these results. The lack of longitudinal data also prevents the assessment of the temporal progression of right ventricular dysfunction and its impact on long-term outcomes. Furthermore, combining CMR findings with other imaging modalities, as well as comprehensive and detailed clinical and laboratory tests, may enhance diagnostic accuracy and provide a more thorough evaluation of biventricular function.

## 5 Conclusion

This study demonstrates that anti-MDA5 Ab+ DM patients exhibit RV subclinical dysfunction, characterized by impaired systolic function. RV-LAS, blood pool T1 mapping, and RVESRI provide valuable tools for early detection and risk stratification in this population. These findings underscore the importance of routine cardiac evaluation in systemic autoimmune diseases and highlight the need for further research to develop targeted interventions for RV dysfunction.

## Data Availability

The raw data supporting the conclusions of this article will be made available by the authors, without undue reservation.
